# Ingestion of a Duck Bone That Lodges in a Jejunal Diverticulum Causing Localized Perforation Requiring Laparotomy and Small Bowel Resection

**DOI:** 10.1002/ccr3.71016

**Published:** 2025-09-24

**Authors:** Nicholas Long, Christos Costa, Safa Al‐Musawi, Ahmed Zainy, Jasim Al‐Musawi

**Affiliations:** ^1^ Department of General Surgery London Northwest NHS Foundation Trust London UK; ^2^ Department of Surgery Imperial Collage London London UK

**Keywords:** acute medicine, critical care medicine, gastroenterology/hepatology, radiology & imaging, surgery

## Abstract

Accidental foreign body ingestion is common, particularly in elderly patients and denture wearers, even when they don't recall ingestion. CT scans are crucial for diagnosing minimally radiopaque foreign bodies and locating perforations. Management lacks standardized guidelines, so treatment decisions must be individualized between operative and conservative approaches.

## 1 Introduction

In clinical practice, the ingestion of foreign bodies is a common occurrence that generally resolves within 7 days as it passes through the GI tract without complications [1]. However, the ingestion of sharp objects poses an increased risk due to the possibility of hollow viscus perforation. Burk et al. suggest that perforation occurs in up to 1% of cases along the gastrointestinal tract [2]. Diagnosing such perforations is often reliant on computed tomography (CT) imaging, as patients frequently fail to recall accidental ingestions. While management typically involves laparotomy, recent case series have highlighted the efficacy of laparoscopic approaches and, in select cases, conservative measures [2, 3].

The objective of this case report is to present a unique case of small bowel perforation resulting from the ingestion of a duck bone and to discuss its clinical presentation, diagnostic investigations, and management strategies as documented in existing literature.

## 2 Case Summary

### 2.1 History

An 84‐year‐old woman presented to our emergency department with a two‐week history of feeling generally unwell, with loss of appetite and a three‐day history of abdominal pain, fever, and intermittent loose stool.

She had an extensive surgical history, including diverticular disease, gallbladder perforation (for which she had an open cholecystectomy), a right hemicolectomy for colon cancer, and a lobectomy for lung cancer.

### 2.2 Examination

On initial presentation, the patient had abdominal tenderness in the left lower quadrant with no guarding or peritonism. Vital signs were normal. Blood tests were normal apart from a C‐reactive protein (CRP) of 24. A urine sample taken by the general practitioner (GP) 3 days prior had grown *Escherichia coli* . Given the confirmed urinary tract infection, the patient was discharged with advice to continue cefalexin as prescribed by the GP.

## 3 Methods

### 3.1 Differential Diagnosis

When the patient first presented with abdominal pain and abnormal CRP, combined with her surgical history, differentials included diverticulitis, a recurrent infection, or a gastrointestinal complication. The confirmed urinary tract infection added complexity, but her unresolved symptoms required further evaluation for other causes, including possible intra‐abdominal pathology.

### 3.2 Investigations

**FIGURE 1:**
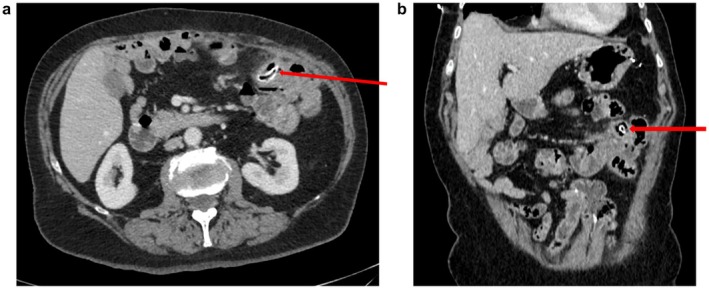
(a) Axial contrast‐enhanced CT image from the initial scan demonstrates a well‐defined, tubular hyperdense foreign body (red arrow) located within a loop of the jejunum in the right mid‐abdomen. The foreign body appears oriented transversely, partially surrounded by intraluminal fluid and gas. There is no definitive evidence of free intraperitoneal air or localized fluid collection to suggest perforation at this stage. The surrounding mesenteric fat shows no stranding, and there is no associated bowel wall thickening or mucosal disruption evident on this initial scan. These findings suggest early or non‐complicated impaction of the foreign body without acute inflammatory changes. (b) Sagittal reconstruction from the initial contrast‐enhanced CT scan, clearly delineating the intraluminal position of the tubular foreign body (red arrow) within a distended jejunal loop. The foreign object is oriented longitudinally in this plane and appears to maintain a fixed position relative to surrounding bowel loops. There is no discernible evidence of transmural extension or perforation. The adjacent mesenteric fat appears preserved without stranding, and the serosal surface of the bowel loop is smooth, further supporting a non‐complicated foreign body impaction at the time of imaging.

Follow‐up was booked in our surgical assessment unit where the patient was seen 3 days later. She had ongoing left lower quadrant pain with guarding, so we proceeded with a contrast‐enhanced abdominal CT scan (see Figure 1a,b). This study showed a tubular, blind‐ending structure intraluminally within the distal jejunal loops on the left side of the abdomen. The structure was radio dense and measured 1.7 cm in maximum length. The jejunal loop itself was thick‐walled, and there was stranding of the adjacent fat. Diverticulosis was noted in the rest of the jejunum. No further imaging such as an ultrasound abdomen was offered at this stage.

**FIGURE 2:**
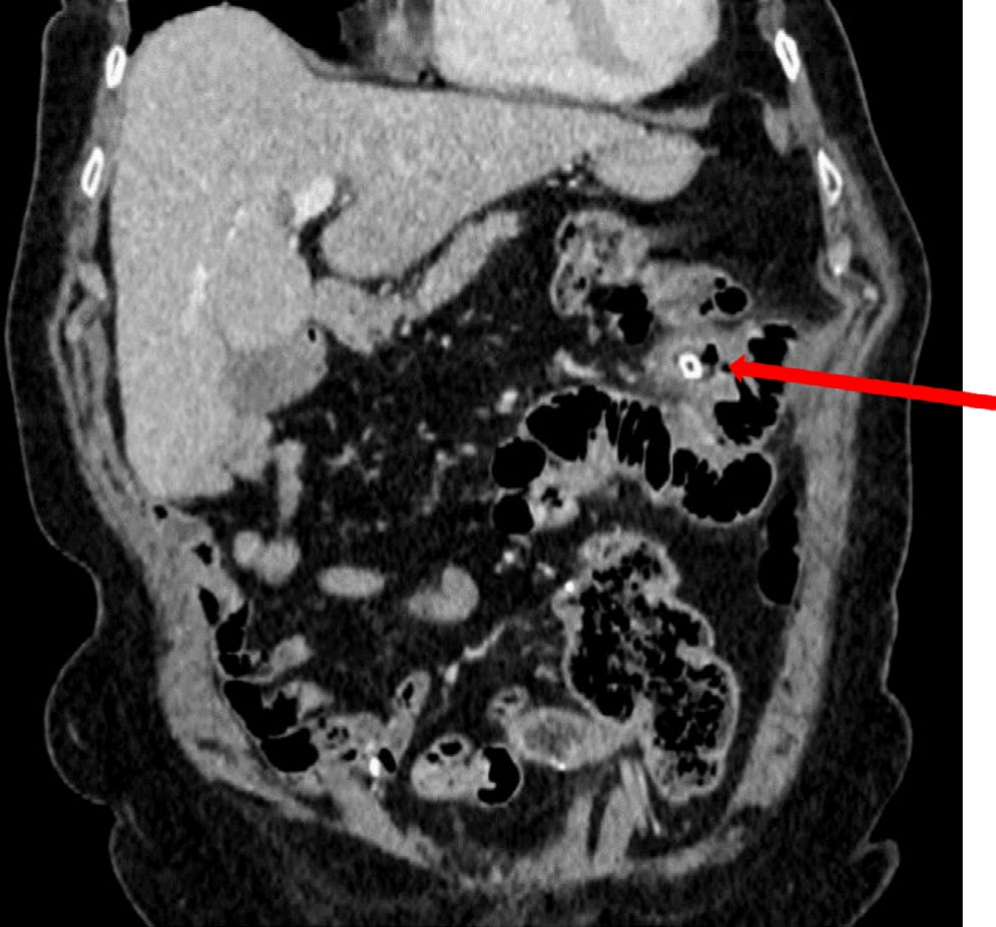
Coronal reconstruction from the follow‐up CT scan performed 2 weeks after the initial study. The tubular hyperdense foreign body (red arrow) remains in a fixed position within a jejunal diverticulum, consistent with impaction. Compared to the initial scan, there is now localized fat stranding and subtle haziness of the surrounding mesenteric fat, indicative of a developing inflammatory reaction. These findings are suggestive of jejunal diverticulitis secondary to the retained foreign body. Notably, the foreign body tip appears to abut or potentially breach the mucosal surface, raising concern for early transmural involvement. The constellation of persistent foreign body impaction, mucosal irritation, and evolving localized inflammation supports the need for surgical intervention to prevent complications such as perforation or abscess formation.

A second contrast‐enhanced CT scan performed 2 weeks later, after the patient re‐presented with worsening abdominal pain, showed that the foreign body previously described was now located inside a jejunal diverticulum, causing uncomplicated diverticulitis (see Figure 2).

### 3.3 Treatment

After the first CT scan, the patient was discharged with safety‐netting advice, hoping that she would naturally pass the foreign body.

On her second presentation, the patient was admitted and treated with ciprofloxacin and metronidazole along with analgesics. Given the foreign body was now lodged in the jejunal wall, we decided to proceed with removal. The patient was initially discussed with the gastroenterology team to ascertain if a double balloon enteroscopy would be feasible to remove the foreign body, but as it was within the bowel wall, there was a risk of perforation, so the decision was made for surgical management.

**FIGURE 3:**
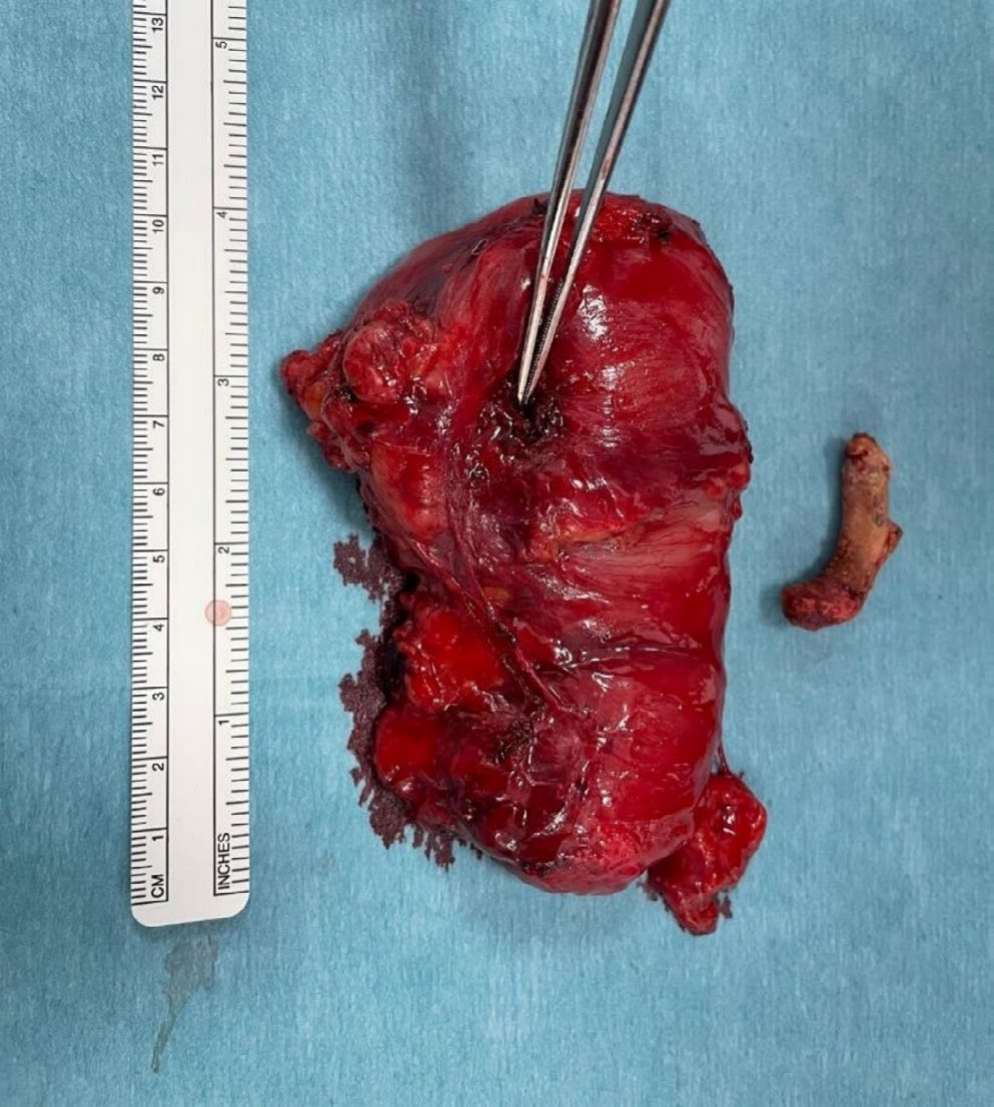
Resected jejunum with the site of localized perforation in the jejunum demonstrated by forceps. The causative duck bone is seen on the right of the image.

Due to her previous abdominal surgeries, it was decided that the best surgical approach was with a laparotomy. Multiple adhesions were resected before running the small bowel. The jejunum was perforated with a localized adhesion to the left side of the abdominal wall. The perforation site was sealed by a small bone (see Figure 3). There was no intra‐abdominal soiling.

The small bowel was resected 10 cm proximal and distal to the site of perforation and rejoined with a side‐to‐side anastomosis.

## 4 Conclusion and Results

Post‐operatively, the patient had a short period of ileus but otherwise recovered well. She was followed up in clinic 6 weeks post‐surgery, and she reported normal bowel motions and no abdominal pain.

## 5 Discussion

GI tract perforations are well cited in the literature. GI perforation from ingested foreign objects occurs in under 1% of cases, and involvement of jejunal diverticula is particularly uncommon, likely due to their broader openings and lower tendency for foreign body entrapment [4]. The cause of perforation can be anything with sharp edges that is easily ingestible. A literature review by Goh et al. showed that 93% of the perforations secondary to ingested foreign bodies are caused by fish bones and other bone fragments [5]. Similarly, Hermosa et al. also suggest that the most common cause of perforation secondary to foreign body ingestion is due to toothpicks and animal bone fragments [6]. Ultimately, the incidence also varies with the dietary habits of each population [7]. Certain factors can increase the likelihood of foreign body ingestion and should raise a clinician's suspicion when a patient presents with nonspecific symptoms. Individuals at higher risk include the elderly, denture wearers, alcoholics, and psychiatric patients [8]. Dentures reduce the tactile sensitivity of the palate, making it harder to detect small objects in the mouth [7, 9], and have been involved in up to 80% of accidental foreign body ingestion cases [4]. In this case report, the patient was indeed a denture wearer.

Another notable aspect investigated in the literature is the site of perforation. Goh et al. reported that 29% of perforations occurred in the anus or distal rectum, with the remaining 71% being intra‐abdominal, most commonly in the distal ileum, accounting for 39% of cases [5]. Similarly, Hermosa et al. found that the colorectal region was the most frequent perforation site (54.5%), followed by the terminal ileum (21.2%) and higher in the small bowel (24.6%) [6].

Typically, patients presenting with intestinal perforation due to an ingested foreign object have typical symptoms and signs of an acute abdomen. These include abdominal pain, peritonitis, nausea and vomiting, gastrointestinal bleeding, abscess development, and intestinal obstruction [8]. Another challenging aspect is that patients often unintentionally fail to disclose the ingestion of a foreign object. This omission, combined with a frequently confusing clinical presentation, can hinder and delay diagnosis. Without this crucial information being mentioned during the consultation, foreign object ingestion is unlikely to be at the top of a clinician's differential diagnosis list [7].

Foreign objects swallowed are infrequently identified on standard X‐rays due to their typically small size and low radiopacity [10], in addition to often being concealed by intestinal gas [7]. A prospective study of Ngan et al. involving more than 300 individuals who ingested fish bones revealed that routine abdominal X‐rays had a sensitivity as low as 32% [11]. Patients, particularly those exhibiting symptoms of bowel obstruction or peritonitis, are likely to undergo an abdominal CT scan, which is crucial for diagnosing potential perforations. CT scans demonstrate high sensitivity in detecting abdominal perforations, ranging from 85% to 95%, according to the literature [11]. While the sensitivity for identifying ingested objects is slightly lower, ranging from 70% to 85% [7, 12]. From these objects, the sensitivity is higher for calcified foreign bodies, such as chicken bones or, in this case, a duck bone. Toothpicks, although non‐calcified, are also detectable on CT scans; however, their detection varies based on the toothpick's attenuation. When ingested, toothpicks are typically dry and air‐filled, resulting in a lower attenuation coefficient, which increases after a few days due to fluid absorption [13]. While diagnosis is generally confirmed with a CT scan, often a re‐evaluation of the scan is required after the patient discloses the potential foreign body ingestion [5, 9]. In instances where a foreign body is suspected but the CT scan results are inconclusive, diagnostic laparoscopy becomes necessary [3, 14].

The significance of a CT scan lies in its increased sensitivity in detecting pneumoperitoneum, a crucial observation especially in cases involving foreign body penetration, thus augmenting its diagnostic efficacy [15]. It is pertinent to note that a non‐contrast CT scan is preferred, as the utilization of oral contrast during the procedure may obscure the visualization of radiopaque foreign bodies. Common CT findings indicative of foreign body presence within the intestines encompass augmented mesenteric fat density, peritoneal gas accumulation, and thickened intestinal walls [7, 8]. Notably, intestinal wall thickening emerges as the predominant observation in existing literature, succeeded by increased mesenteric fat density [10]. In rare occurrences, foreign bodies can precipitate bowel obstruction, clinically manifesting through characteristic CT features such as dilated proximal bowel loops, a small bowel diameter surpassing 2.5 cm, a discernible transition point denoting a shift from normal to abnormal bowel caliber, and bowel wall thickening [16]. Furthermore, gastrointestinal bleeding may ensue as a rare complication stemming from foreign body‐induced erosion of intestinal walls into adjacent blood vessels. Instances of acute appendicitis and Meckel's diverticulum attributable to foreign body impaction are infrequent, although documented cases exist within the literature [7, 17].

Treatment strategies hinge on the foreign body's location, timing of presentation, and associated complications like perforation, hemorrhage, or obstruction. Endoscopic removal is preferred for esophageal or gastric foreign bodies presenting early, while surgical intervention, typically segmental resection, is indicated for those in the small intestine [7, 18]. In a few asymptomatic cases, conservative management remains a viable option [19]. Management of small bowel perforation, like his case, due to ingested foreign bodies lacks definitive guidelines, necessitating a case‐by‐case approach considering patient symptoms, clinical observations, and imaging findings. While early removal via endoscopy is feasible for larger objects, its efficacy for smaller items like bones is debatable [14]. Though emergency laparotomy is customary for proven perforation and peritonism, Yang et al. and Chia et al. showcased successful laparoscopic approaches for removal [4, 20]. Conservative management remains viable in certain instances [6].

## Author Contributions

**Nicholas Long:** data curation, formal analysis, writing – original draft, writing – review and editing. **Christos Costa:** conceptualization, data curation, formal analysis, writing – original draft, writing – review and editing. **Safa Al‐Musawi:** data curation, writing – review and editing. **Ahmed Zainy:** writing – review and editing. **Jasim Al‐Musawi:** supervision.

## Consent

A written informed consent form has been obtained from the patient according to the journal guidelines.

## Conflicts of Interest

The authors declare no conflicts of interest.

## Data Availability

Data openly available in a public repository that issues datasets with DOIs.
